# In vivo CRISPR knockout screen identifies Polr1a as a key driver and a potential therapeutic target for melanoma metastasis

**DOI:** 10.1038/s41388-026-03851-4

**Published:** 2026-06-17

**Authors:** Anna F. Fajardo, Chethana P. Gowda, Emily Johnson, Ricardo Petroni, Vivek S. Tomar, Zhenqiu Liu, M. Andres Blanco, Jacob Janssen, Irina A. Elcheva, Matthew Lanza, Serge Y. Fuchs, Vladimir S. Spiegelman

**Affiliations:** 1 College of Medicine, Pennsylvania State University, 500 University Drive, Hershey, PA USA; 2 Department of Biomedical Sciences, University of Pennsylvania, Philadelphia, PA USA; 3 Present Address: Department of Statistics, Radiation Effects Research Foundation, Hiroshima, Japan

**Keywords:** Metastasis, Melanoma

## Abstract

Identification and characterization of novel mechanisms driving melanoma metastases and ways to target them are paramount for the development of effective treatment modalities. Here, we employed in vivo CRISPR knockout screening targeting the genes associated with poor prognosis to identify Polr1a as a potent driver of melanoma metastasis. High Polr1a levels correlate with increased metastasis and reduced survival in patients. Polr1a inhibition suppressed migration, invasion, and the ability of melanoma cells to colonize lungs. Ribo-seq analysis revealed that Polr1a is involved in regulating the non-canonical NF-κB pathway. Indeed, targeting Polr1a decreased levels of RelB and p52 and suppressed non-canonical NF-κB transcriptional activity; this suppression was responsible for the effects of Polr1a on melanoma cell migration. Accordingly, pharmacological inhibition of Polr1/Polr1a suppressed cell migration, tumor growth, and metastases. We discuss the potential utilization of Polr1 inhibitors for neoadjuvant treatment of melanoma.

## Introduction

Metastatic melanoma is an extremely aggressive disease with a very high dissemination rate and a five-year survival rate of less than 15% [1, 2]. Current treatment strategies for metastatic melanoma include neoadjuvant/adjuvant immunotherapy (IL-2, anti-PD1, Anti-CTLA4 therapy), BRAF and MEK inhibitors, cytotoxic chemotherapy, radiation therapy, and a combination of these approaches. However, not all patients respond to these therapies, and survival rates are still quite low, which shows a continued need for new approaches and the discovery of novel metastasis drivers [3, 4].

Metastasis is a multi-stage process that includes, among others, epithelial-mesenchymal transition (EMT), invasion, intravasation, survival in the bloodstream, premetastatic niche formation, micrometastasis, and metastatic colonization. Major progress has been achieved in recent years to advance our understanding of the mechanisms that govern different stages of melanoma [5]; however, our understanding of melanoma metastasis is still far from complete.

CRISPR-based loss- and gain-of-function screens have become the gold standard for identifying genes that drive therapy resistance, immune evasion, and metastasis [6–9]. In vivo CRISPR screening approaches have successfully uncovered novel metastatic regulators in several cancer types [10–12], but their application is limited by the technical challenges associated with maintaining adequate representation of large, genome-wide libraries in animal models.

To address these limitations and enhance clinical relevance, we developed a focused in vivo CRISPR knockout screening strategy targeting genes whose expression is associated with poor prognosis in melanoma patients. Using this curated library in syngeneic melanoma models, we compared sgRNA representation between primary tumors and lung metastases to identify genes specifically required for metastatic progression.

This approach revealed Polr1a, a core RNA polymerase I subunit, as a novel driver of melanoma metastasis. RiboSeq analysis revealed that Polr1a inhibition downregulates non-canonical NF-κB signaling. Polr1a inhibition significantly reduced melanoma cell migration and invasion in an NF-κB-dependent manner, while pharmacological targeting of Polr1 demonstrated strong anti-tumor and anti-metastatic effects in vivo. To facilitate evaluation of neoadjuvant therapeutic strategies, we also established a novel spontaneous melanoma metastasis model incorporating primary tumor resection using SW1 cells in C3H mice.

## Materials and methods

### Gene selection for custom CRISPR KO library

The TCGA melanoma gene expression dataset was obtained from the UCSC Xena browser (https://xenabrowser.net/datapages/, accessed on 02/20/2021) [13]. RNA sequencing was performed using the Illumina HiSeq 2000 platform by the University of North Carolina TCGA Genome Characterization Center. The dataset includes 474 samples and 20,531 genes, along with clinical metadata such as overall survival. Gene expression values were provided as RSEM-normalized counts and log₂-transformed for analysis.

We conducted univariate Cox regression analyses for each gene in the TCGA melanoma dataset using the survival R package (https://github.com/therneau/survival). A total of 348 candidate genes with *P*-values < 0.05 were selected (Table S1). For each gene, we report the estimated coefficient (coef), hazard ratio for a one-unit increase in gene expression (exp(coef)), standard error (SE), z-score, and corresponding *P*-value. The false discovery rate (FDR), calculated using the Benjamini–Hochberg procedure, is also included. A positive coefficient indicates a higher hazard ratio and worse overall survival, while a negative coefficient suggests a lower hazard ratio and better survival.

### CRISPR KO screen

The custom CRISPR KO library was designed to target genes associated with the poor prognosis of melanoma patients. Each gene was targeted by 6 sgRNAs; the pool of non-targeting RNAs consisted of 114 sgRNAs (~5% of all sgRNAs) (Table S2). gRNAs were cloned into the pLentiGuide backbone. Library amplification, cells’ transduction, gDNA isolation, amplification, and sequencing were performed as previously described [14]. Briefly, 2.5 μg of pooled library plasmid were electroplated into 25 μL of Endura electrocompetent cells and grown overnight on LB/agar plates. Then, plasmids were purified using EndoFree Plasmid Maxi Kit (Qiagen, 12362, Venlo, Netherlands). For virus production, 70% confluent HEK293T cells were transfected with 10.71 μg of pooled CRISPR KO library, 5.35 μg of psPAX2, and 2.68 μg of pMD2.G with polyethyleneimine PEI MAX (Polysciences, 24765-100, Warrington, PA, USA). The virus was collected after 48 h, filtered through a 0.44 μm filter, concentrated using Amicon Ultra 15 (Millipore Sigma, Burlington, MA, USA) centrifugal filters, aliquoted, and frozen at –80 °C until use. Virus titer was estimated using a colony formation assay. 5 M of SW1 cells were transduced with 0.3 MOI of CRISPR KO library to achieve 500x coverage. After 24 h, the medium was replaced with a fresh one, and after 48 h, 1 μg/mL of puromycin was added for selection. When selection was completed, 5 M cells were collected for gDNA isolation, and the rest were injected subcutaneously into C3H mice (1 M cells/mouse). Tumors grew for 38 days, after which the tumor and lung metastasis samples were harvested.

### Sequencing and analysis of CRISPR screens

gDNA was isolated from at least 5 M cells, tumor, and lung metastasis samples using DNeasy Blood & Tissue Kit (Qiagen, 69504). gDNA equivalent of 500x library coverage was amplified using Hifi high fidelity polymerase (Stellar Scientific, RAD HF1100, Baltimore, MD, USA), and primers synthesized in IDT according to previously described sequences [14] (Table S4). Each PCR reaction contained 500 ng gDNA, 0.5 μL HiFi high fidelity polymerase, 10 μL of 5x buffer, 2 μL of forward and reverse primers and molecular biology grade water up to 50 μL. A total of 8000 ng was amplified for each sample. PCR conditions were as follows: 1 min at 95 °C, followed by 15 s at 95 °C, 15 s at 65 °C, 30 s at 72 °C repeated for 25 cycles and finally 5 min at 72 °C. Finally, PCR products were purified with Agencourt AMPure XP SPRI beads according to the manufacturer’s instructions (Beckman Coulter, A63880, Brea, CA, USA). Product purity was evaluated using Agilent 2100 BioAnalyzer and DNA 1000 Kit (Agilent Technologies, 5067-1504, Santa Clara, CA, USA), and concentration was measured with Qubit™ 1X dsDNA HS Assay (Qubit, Q33230, Quebec City, Canada). Libraries were sequenced in the Penn State College of Medicine Genome Sciences core (RRID:SCR_021123) on Illumina NovaSeq 6000 (Illumina, San Diego, CA, USA), to get on average 1.5 million, single-end 61 bp reads to achieve >500X coverage.

For bioinformatic analysis, the Galaxy platform was used [15]. 5’ adapters (TTGTGGAAAGGACGAAACACCG) were trimmed from the reads. Gene ranking was performed using the MAGeCK algorithm. Counts were normalized on non-targeting sgRNAs. The top 25 enriched or depleted sgRNAs were identified in metastasis samples versus tumors.

### In vivo experiments

Male and female C3H/HeJ mice (6–8 weeks old) from Charles River and NSG mice (6–8 weeks old) from Jackson Laboratories were used for subcutaneous injection of SW1/451Lu cells, respectively. 7 × 10^5^ cells (SW1) or 2 × 10^6^ cells (451Lu) were resuspended in phosphate-buffered saline (PBS, Corning, NY, USA) and injected into the right flank of mice. Group sizes were chosen based on the literature data and previous laboratory experience. Tumor size was measured two times a week using calipers, and tumor volume was calculated based on the following formula: volume = length [mm] × width^2^ [mm^2^]/2. When tumor size reached 90–100 mm^3^, mice were randomly assigned to the CX-5461 or vehicle treatment groups (*n* = >8) using RandoMice software [16]. The investigator was not blinded to the group allocation during the experiment. CX-5461 (batch #04) was reconstituted in sodium phosphate buffer, pH=4.5 and injected intraperitoneally at 50 mg/kg two times a week (C3H mice) or via oral gavage once every 5 days (NSG mice). Treatments were started when the tumor size reached 120 mm^3^ and were continued through the endpoint (day 38 after SW1 cell injection or day 42 after 451Lu cell injection). Treatment conditions were selected based on previously published data, which showed that 50 mg/mg injected i/p or via oral gavage did not cause toxicity in mice, did not affect the body mass and general health condition [17, 18]. At day 38/42 mice were sacrificed, lung macro metastases were counted, and tissue was fixed in formalin for further H&E staining.

All experiments were approved by the local IACUC committee.

### Statistical and image analysis, reproducibility

Data analysis was carried out using Microsoft Excel (Microsoft Corporation, USA) and GraphPad Prism (GraphPad Software, USA) software. Data are presented as the group arithmetic mean (M) and standard deviation (SD) of at least three biological replicates. Most of the in vitro experiments with genetic Polr1a inhibition were repeated using 2 different shRNA constructs; only one is shown in all pictures. Criteria for statistical comparison were selected based on data distribution and the number of samples. Shapiro-Wilk or Kolmogorov-Smirnov tests were used to test distribution normality, and a corresponding statistical test was applied further to detect the statistically significant differences. One-way ANOVA, Student t-test, and Mann-Whitney tests were used; all tests were two-tailed. No assumption about equal SD between the groups was applied. Tukey adjustment was applied for pairwise comparisons using one-way ANOVA. Differences were considered statistically significant at *p* < 0.05. Sample sizes were chosen based on the literature data and previous laboratory experience to reach a power of 80%. Western blot experiments were reproduced at least two times; representative pictures are shown. For all proliferation and migration data measured with IncuCyte, representative graphs from one experiment with at least three biological replicates are shown as mean ± SD; experiments were repeated 2 to 4 times. In the Figure legends, “n” represents the number of technical replicates, and “N” corresponds to the number of biological replicates.

Image analysis was performed using the ImageJ software 1.54 f or Fiji.

Additional detailed Materials and methods can be found in the Supplemental Materials.

## Results

### CRISPR KO screening identifies Polr1a as a potential driver of melanoma metastasis

To identify new drivers of metastasis, we have designed a custom CRISPR loss-of-function library targeting specific genes that we determined as being associated with poor prognosis in melanoma patients using bioinformatic analysis (Table S1). This library included 6 sgRNAs targeting each of 348 selected genes and 114 ( ~ 5% of all sgRNAs) non-targeting sgRNAs (Table S2) cloned into the pLentiGuide backbone. Then, we generated a stable SW1/Cas9 cell line expressing Cas9 (Fig. S1A) as our CRISPR library was designed as a two-vector system. To ensure the same level of Cas9 activity in all the cells used for the CRISPR screen, we have selected single-cell clones, tested them in the GFP-Cas9 activity assay using the pXPR-11 plasmid, and selected clone 5 for subsequent CRISPR screen as the one having the most active Cas9 (Fig. S1B, C). Although SW1/Cas9 cells showed decreased tumor growth, they exhibited metastatic ability comparable to parental SW1 cells (Fig. S1D–F).

**Fig. 1:**
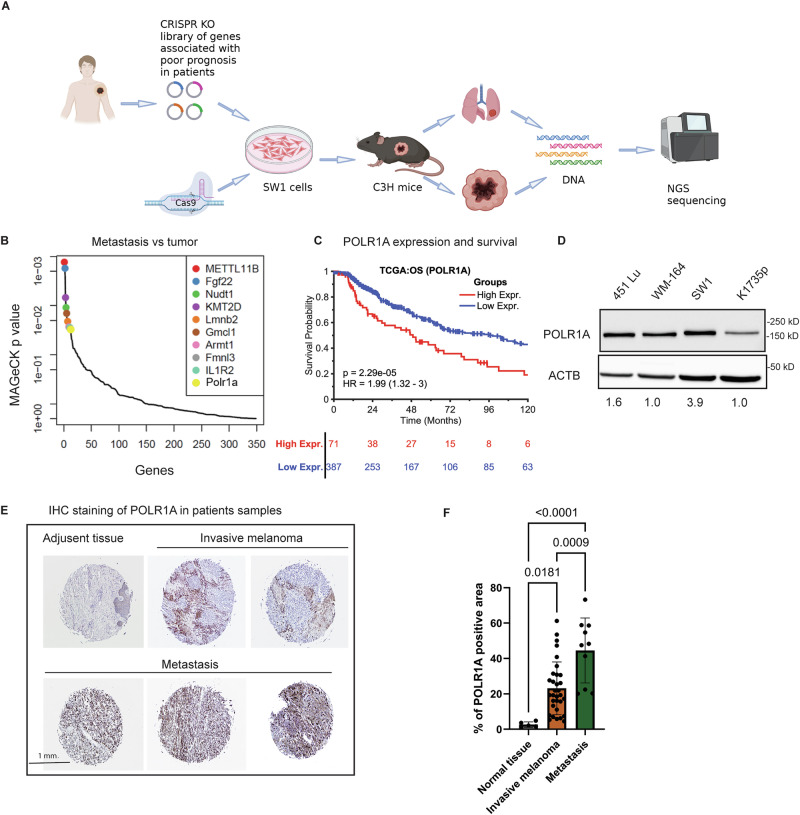
In vivo CRISPR knockout screen identifies Polr1a as a metastasis driver. **A** Workflow of CRISPR knockout in vivo screen to identify the potential targets for metastasis regulation. **B** Top depleted genes in lung metastasis versus tumors in the CRISPR KO screen. **C** Survival curve showing that induced expression of POLRIA correlates with lower overall survival in patients with melanoma, *P* < 0.005. **D** Western blot of Polr1a content in the non-metastatic (K1735p and WM-164) versus metastatic (SW1 and 451 Lu) melanoma cell lines. The numbers under the ACTB bands represent the quantification of the ratio of Polr1a content in metastatic/ non-metastatic cell lines. **E** IHC staining of POLRIA in normal human skin tissue, invasive melanoma tissue, and metastasis (representative samples from TMA are shown). **F** Quantification of human melanoma microarray stained with IHC for POLRIA expression (one-way ANOVA, normal tissue = 5, invasive =32, metastasis=10).

To perform an in vivo screen, we transduced enough SW1/Cas9 cells to achieve 500x sgRNA coverage with 0.3 MOI of CRISPR KO library and selected cells with puromycin. 10 days after library transduction, 1 M cells (500X coverage) were injected subcutaneously, and 1 M cells were harvested as a pre-inoculation sample. 38 days after tumor inoculation, tumors and lung metastases were harvested, with individual lung metastases being excised and pooled together, and all samples were processed for next-generation sequencing (Fig. 1A). We first examined global characteristics of the sequencing results. As anticipated, read count distributions varied significantly between samples. The Gini index was 0.1088 in the pre-implementation sample, indicating even coverage in the library at the start of the in vivo experiment. However, Gini indices rose to 0.6406 in the tumor sample and 0.6837 in the metastatic lesions sample (Fig. S1G). This suggests that marked clonal competition and evolution occurred over the course of tumor growth and continued slightly during the metastatic process. We then used MAGeCK analysis to reveal sgRNAs enriched or depleted in the metastasis compared to tumor samples. Among the identified top hits depleted from the metastasis sample, there were genes with already well-established roles in metastasis and tumorigenesis, such as KMT2D and LMNB2 (Fig. 1B), confirming the validity of this screen. Some top-scoring sgRNAs showed inconsistent enrichment trends and were excluded from further validation despite scoring significantly. From the top candidates, the Polr1a gene was selected for further validation because of its recognized cellular function and unknown role in melanoma metastasis.

### High levels of POLR1A expression correlate with higher metastatic potential and lower survival in patients

To assess the clinical relevance of our findings, we examined the association between POLR1A expression and overall survival in melanoma patients using the log-rank test with TCGA data. Gene expression and corresponding clinical data were obtained from the UCSC Xena browser (https://xenabrowser.net/datapages). The TCGA melanoma cohort included a total of 473 patients with cutaneous primary and metastatic tumors, among whom 458 had both clinical information and RNA-seq expression data available. Of these, 105 cases were primary melanomas, and 353 were metastatic melanomas. Overall survival (OS) data for these patients were originally curated by Liu et al. [19].

We evaluated the prognostic significance of POLR1A expression in both the full cohort of 458 melanoma patients (Fig. 1C) and the subset of 353 metastatic melanoma patients (Fig. S1H). High POLR1A expression was significantly associated with poorer overall survival compared to low expression in both the full cohort and the metastatic subset, with a more pronounced effect observed among patients with metastatic disease (Fig. S1H,I).

Assessment of sibling pairs of melanoma cell lines that differ in their metastatic potential revealed a substantial increase in Polr1a level in SW1 and B16F10 mouse cells in comparison with non-metastatic K1735p and B16F1, respectively (Figs. 1D; S1J). Similarly, 451Lu human metastatic cells exhibited higher Polr1a levels than their parental poorly metastatic cell line WM-164 (Fig. 1D).

IHC analysis of human melanoma tissue microarray revealed that invasive melanoma samples had significantly higher Polr1a expression in comparison with normal adjacent tissue, and Polr1a expression levels in metastatic samples were further significantly elevated in comparison with the invasive tumors (Fig. 1E, F). Overall, these results show higher levels of POLR1A in metastatic melanoma and its association with poor overall survival, further supporting our hypothesis that Polr1a plays a significant role in melanoma metastasis.

### Polr1a stimulates migration and invasion of melanoma cells

At least two specific Polr1 (CX-5461) and Polr1a (BMH-21) inhibitors were developed and implemented previously in preclinical cancer models [17, 20–23]. In addition, CX-5461 is currently used in two stage 1 clinical studies in patients with solid tumors (NCT04890613; NCT06606990), one clinical trial stage 1 has already been completed (NCT02719977), and CX-5461 was shown to be well tolerated at therapeutic doses [24]. However, these small molecules were not tested for their potential anti-metastatic activities. To investigate the role of Polr1a in melanoma metastasis, we evaluated the in vitro effect of pharmacological inhibition on the proliferation and migration rates of cells.

**Fig. 2:**
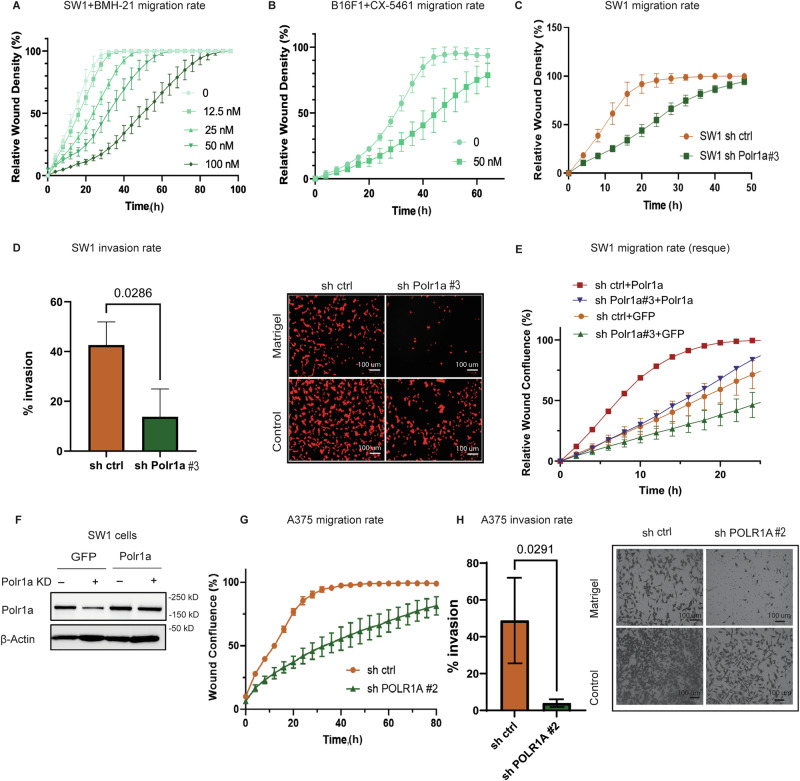
In vitro validation of CRISPR KO screen results. **A** SW1 cells migration rate measured with the IncuCyte live cell analysis imaging system after 24 h pretreatment with corresponding doses of BMH-21 (*n* ≥ 3, *N* = 3). **B** B16F1 cells migration rate measured with the IncuCyte live cell analysis imaging system after 24 h pretreatment with corresponding doses of CX5461(*n* ≥ 3, *N* = 3). **C** SW1 cells’ migration rate measured with the IncuCyte live cell analysis imaging system. (*n* ≥ 3, *N* = 3). **D** Quantification of invasion of SW1 cells with Polr1a KD #3 (Mann-Whitney test, *n* = 4/group, *N* = 3) and representative pictures of cells invaded through the matrigel or migrated through the control inserts. **E** SW1 cells with re-expressed Polr1a migration rate measured with IncuCyte live cell analysis imaging system. (*n* ≥ 3, *N* = 3). **F** Western blot showing representative protein levels of Polr1a re-expressed in sh ctrl or sh Polr1a #3 SW1 cells. **G** A375 cells’ migration rate measured with the IncuCyte live cell analysis imaging system. (*n* ≥ 3, *N* = 3). **F** Quantification of the invasion rate of A375 cells and representative pictures. (Unpaired t test, t = 3.330, df=4, *n* = 3/group).

The same doses of BMH-21 caused much smaller effects on proliferation (Fig. S2A, B) compared to migration rate (Figs. 2A; S2C, D): whereas 50 nM of BMH-21 just slightly inhibited SW1, B16F1 proliferation, it dramatically impaired the migration ability. The same effect was observed with CX-5461 treatment: while 50 nM just slightly inhibited the proliferation of B16F1 (Fig. S2E) and SW1 (Fig. S2F), it had a strong effect on the migration rate (Figs. 2B; S2G).

To further investigate the effects of Polr1a on tumorigenic properties of melanoma cells, we used the shRNA-mediated knockdown of Polr1a (shPolr1a) that efficiently decreased Polr1a protein levels (Fig. S2H), but did not affect viability (Fig. S2I), colony formation (Fig. S2J), anchorage-independent growth (Fig. S3A) and proliferation (Fig. S3B, C) of mouse melanoma cells. However, migration of SW1 (Fig. 2C) and B16F1 (Fig. S3D) mouse melanoma cells was severely impaired. Moreover, Polr1a KD led to an over 2-fold reduction in invasion of SW1 cells compared to the non-targeting shRNA control (Fig. 2D). Ectopically expressing Polr1a in the SW1 cells with Polr1a KD rescued the migration phenotype and promoted the migration rate of sh ctrl cells (Fig. 2E, F).

To support our observations in mouse melanoma cells, we generated doxycycline-induced POLR1A KD in human melanoma cells (Fig. S3, F) to investigate its impact on migration and invasion. Reduction in POLR1A level caused a notable decrease in migration (Figs. 2G; S3G, I) and invasion (Figs. 2H; S3H, J) of A375 and 451 Lu cells.

These data suggest that Polr1a downregulation particularly decreases migration and invasion of melanoma cells.

### Polr1a regulates migration in an NF-κB-dependent manner

**Fig. 3:**
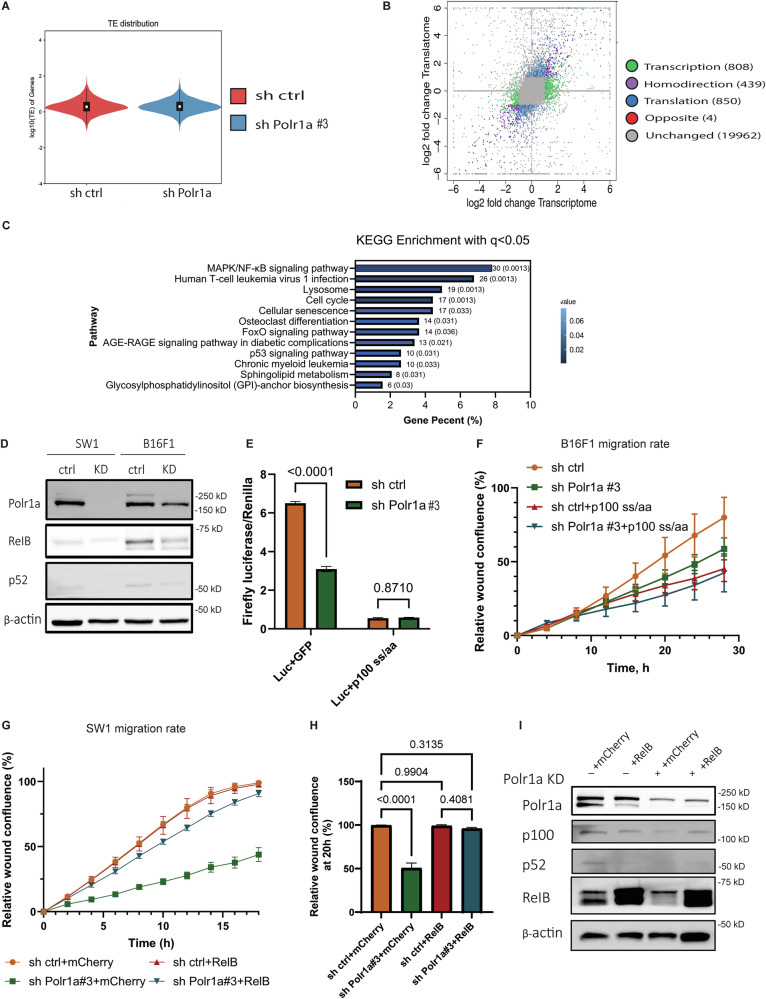
Polr1a regulates the non-canonical NF-κB signaling pathway. **A** TE distribution violin map in each group, **B** Scatter plot of genes affected by Polr1a KD classified in each direction of differences in translation and transcription level based on joint analysis of RNA-seq and Ribo-seq data. Shown subgroups of genes are: “Transcription”: regulated only on transcription level (808 genes), “Translations”: regulated only on translation level (850 genes), “Homodirection”: regulated both on transcription and translation levels (439 genes), “Opposite”: having opposite trends in transcription and translation [4] and unchanged (19 962 genes). **C** KEGG enrichment bar chart of genes affected by Polr1a KD on translation level **D** Representative western blot showing protein levels of RelB and p52 in SW1 and B16F1 cells with Polr1a KD (*N* = 2) **E** NF-κB activity measured using NF-kB reporter system and dual luciferase assay in B16F1 cells with Polr1a KD, transduced with either WT p100 or mutated p100 ss/aa (2-way ANOVA, *n* = 3, *N* = 3) **F** B16F1 cells migration rate measured with IncuCyte live cell analysis imaging system (*n* = 4, *N* = 2) **G** SW1 cells with re-expressed RelB migration rate measured with IncuCyte live cell analysis imaging system (*n* = 4, *N* = 3). **H** Quantification of (**G**) at the 20 h timepoint (ordinary one-way ANOVA, *n* = 4). **I** Representative western blot showing the levels of p100, p52, and RelB in the cells used for migration assay in (**G**) (*N* = 2).

Consistent with the established role of Polr1a in transcription of ribosomal RNA, knockdown of Polr1 led to decreased Polr1-mediated transcriptional activity in SW1 cells (Fig. S4A). However, to our surprise, Ribo-seq analysis of SW1 cells upon Polr1a KD revealed no change in global translation efficiency (Fig. 3A), and the amount of 28S and 18S rRNA also did not change in shPolr1a cells (Fig. S4B). These results suggest that effects on translation of specific mRNA, rather than global translation, are likely responsible for the observed Polr1a-mediated phenotypes in melanoma cells. To further investigate this specific effect and the potential pathways regulated by Polr1a we performed a joint analysis of RNA-seq and Ribo-seq data and divided all genes into 5 groups: regulated only on transcription level (808 genes), translation level (850 genes), both transcription and translation levels (439 genes), having opposite trends in transcription and translation [4] and unchanged (19,962 genes) (Fig. 3B). KEGG enrichment pathway analysis revealed the MAPK/ NF-κB signaling pathway as the top hit affected on the translational (Fig. 3C) level by Polr1a KD. Closer evaluation of the genes regulated by Polr1a within MAPK/ NF-κB signaling revealed specific enrichment of genes belonging to the non-canonical NF-κB signaling pathway (Fig. S4C).

To validate the finding that Polr1a regulates the non-canonical NF-κB pathway, we performed polysome profiling of SW1 cells with subsequent analysis of NF-κB and RelB mRNA % in each monosome and polysome fraction, and confirmed that NF-κB2 and RelB translation is downregulated in the cells with Polr1a KD (Fig. S4D). Then we analyzed the levels of RelB and p52 proteins in SW1 and B16F1 cells and observed that they were downregulated in the cells with Polr1a KD (Fig. 3D). Polr1a KD caused a 2.1-fold decrease in the NF-κB transcriptional activity of B16F1 cells estimated using the luciferase reporter. To efficiently suppress non-canonical NF-κB signaling, we expressed mutant p100, which has a serine to alanine amino acid substitution (p100^ss/aa^) that prevents effective phosphorylation of p100 by IKK_α,_ which in turn blocks activation of non-canonical NF-κB [25]. Ectopic expression of p100^ss/aa^ resulted in a dramatic decrease of NF-κB activity, and that activity could no longer be regulated by Polr1a (Fig. 3E). The finding that Polr1a KD decreased NF-κB activity was confirmed in SW1 cells using another NF-κB reporter system (Fig. S4E).

To strengthen the causality argument, we induced POLR1A KD in 451Lu and A375 cells and demonstrated that p100 and RelB protein levels diminished in parallel with POLR1A reduction (Fig. S5A). Furthermore, Polr1a KD in mouse SW1 and human A375 and 451Lu melanoma cells also resulted in the downregulation of mRNA expression of target genes of the non-canonical NF-kB pathway, such as *TNFSF13B*, *TRAF3*, and Cxcl-13 (Fig. S5D, E).

Finally, we evaluated the effects of blocking the non-canonical NF-κB pathway on the migration properties of B16F1 cells. While cells with Polr1a KD had impaired migration ability in comparison with control, overexpression of mutant p100 caused inhibition of migration and negated the effect of Polr1a on the rate of migration. (Figs. 3F; S4F). Reciprocally, overexpression of transcriptional activator of non-canonical NF-κB signaling, RelB, alone or in combination with p100 (Figs. 3G–I and S5B, C), rescued the migration phenotype induced by Polr1a KD. To further evaluate possible NF-κB pathway downstream targets directly affecting migration properties, we assessed the epithelial-mesenchymal transition markers and found that vimentin and Slug were downregulated both in mouse and human Polr1a KD cells (Fig. S5F). These data further substantiate our findings that Polr1a regulates migration properties through the non-canonical NF-κB pathway.

### Polr1a KD impairs the metastatic potential of melanoma cells in vivo by reducing their lung colonization ability

**Fig. 4:**
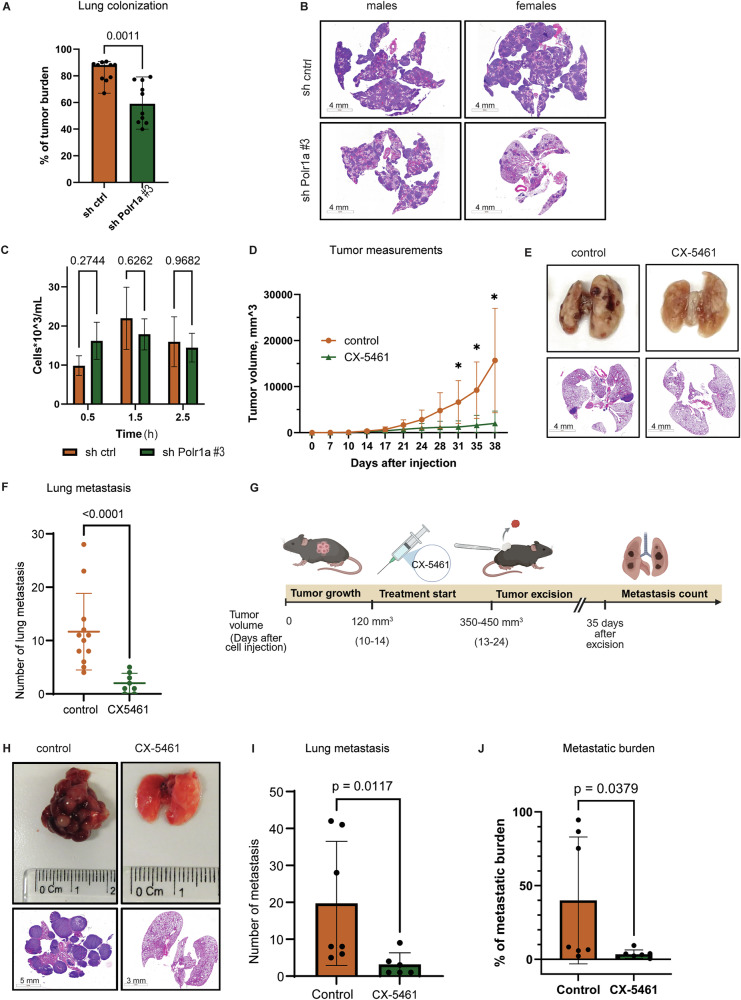
Polr1a inhibition as a therapeutic strategy. **A** Quantification of total area of metastatic burden in lungs of mice IV injected with SW1 cells with Polr1a KD (Mann-Whitney test, *n* = 10/group). **B** H&E staining of lungs quantified in (**A**). **C** Number of CTC in mice IV injected with SW1 cells with Polr1a KD (2-way ANOVA, *n* = 4/group). **D** Tumor growth dynamics in C3H mice treated with CX-5461 (*n* ≥ 8 per group). Data are represented as mean ± SD. *p* values are comparing the control group vs CX-5461-treated group by unpaired t test with Welch correction. *P < 0.05. **E** Pictures of lungs with metastasis on day 37 and H&E-stained lungs. **F** Number of macrometastases in mice treated with CX-5461 (Mann-Whitney test, ≥ 8 per group). **G** Scheme of experiment to evaluate the effect of CX-5461 treatment on lung metastasis in the absence of the primary tumor. **H** Pictures of lungs with metastasis on day 35 after surgery and H&E-stained lungs. **I** Number of macrometastases in mice with resected tumors treated with CX-5461 (Mann-Whitney test, *n* = 7). **J** Quantification of tumor burden in the lung tissues (Mann-Whitney test, *n* = 7).

To further investigate the role of Polr1a in the metastatic potential of melanoma cells, we used the experimental metastasis model. Mice IV injected with SW1 cells with Polr1a KD had significantly lower metastatic burden in the lung tissue compared to the control (Figs. 4A, B; S6A). To discriminate the probability of Polr1a affecting the tumor cells’ survival in the bloodstream, we IV injected sh ctrl and sh Polr1a cells expressing luciferase and collected blood after 0.5 h, 1.5 h and 2.5 h. Polr1a KD did not significantly change the numbers of circulating tumor cells (CTC) in the bloodstream (Fig. 4C), suggesting that Polr1a may regulate later stages of the metastatic process, such as extravasation, homing and colonization of distant sites, but not survival of CTC in the bloodstream.

### Polr1a is a potential therapeutic target for metastatic melanoma treatment

To evaluate the effect of Polr1 inhibition on spontaneous melanoma metastasis, we inoculated SW1 cells subcutaneously into the syngeneic C3H mice to generate the tumors and treated mice with CX-5461 (in previously-reported non-toxic doses [26]). CX-5461 significantly slowed down the tumor growth (Fig. 4D) and decreased final tumor mass and size (Fig. S6B, C). Finally, we observed a striking decrease in the number of lung metastases in mice treated with CX-5461 (Fig. 4E, F). To confirm this observation with human melanoma cells, we treated NSG mice bearing 451Lu tumors with CX-5461. CX-5461 significantly decelerated tumor growth and decreased the tumor masses (Fig. S6D, E). Only 20% of control mice developed lung micrometastases; however, there were no micrometastases detected in the CX-5461 group (Fig. S6F). These findings indicate that CX-5461 efficiently inhibits melanoma progression.

To confirm that the observed effect of Polr1 pharmacologic inhibition on metastasis is due to specific inhibition of bona fide metastatic properties, not just due to the inhibition of primary tumor growth, we performed survival surgeries to resect primary tumors and then analyzed lung metastases (Fig. 4G). CX-5461-treated mice did not demonstrate a decrease in body mass, which confirms the good tolerance to the drug (Tables S6; S7) and had a significantly lower number of lung metastases (Fig. 4H, I). Also, CX-5461 treatment resulted in significantly lower lung masses, evidencing the absence of the massive metastatic burden that was observed in the control group (Fig. S6G). This observation was confirmed using the quantification of H&E-stained lung specimens, which demonstrated a significant decrease in the surface area of lung tissues occupied by metastases (Fig. 4J). Thus, Polr1 inhibition specifically affects metastatic spread, independent of the effect on the primary tumor. These results demonstrate that CX-5461 exhibits a potent anti-metastatic effect. Observed in all in vivo experiments, high variability in the tumor sizes and number of lung metastases is consistent for the SW1 spontaneous metastasis model in our experience. Despite the unified genetic background of C3H mice, the immune context varies from animal to animal. Due to the complexity and multiple stages involved in the spontaneous metastasis process, metastasis counts always exhibit significant biological variation. However, this does not prevent the achievement of statistically significant differences. Finally, we assessed the immune context of tumors treated with CX-5461 and observed a trend toward reduced CD8⁺ infiltration along with a significant decrease in PD-L1 expression (Fig. S6H).

All together, these results establish a promising basis for the use of Polr1 inhibition as a strategy for the treatment of melanoma metastatic disease.

## Discussion

Metastatic melanoma remains a disease with low survival rates, so there is an urgent need to investigate molecular mechanisms driving metastasis to find new treatments. CRISPR screens emerged in recent years as a powerful tool for discovering novel genes that drive tumor progression. Despite all the advantages of CRISPR screens, certain limitations are making it challenging to use genome-wide CRISPR libraries in combination with spontaneous melanoma metastasis models to identify drivers and suppressors of metastasis. To achieve the necessary sgRNA representation, several million melanoma cells need to be transplanted to one animal, which is technically impossible due to the very fast pace of tumor growth and the prolonged time necessary for the tumor to metastasize to a distant site. Pooling of samples from several animals is one of the commonly used strategies to overcome this challenge [27]. However, applying it for the discovery of metastasis drivers makes it impossible to make an exact comparison of sgRNAs enriched or depleted in metastasis compared to the tumor of the same animal. To overcome this challenge, smaller-sized libraries targeting specific sets of genes can be used. We performed a novel CRISPR screen targeting the genes selected based on the correlation of their expression levels with clinical outcomes. Several genes with established roles in metastasis (i.e., Kmt2d [28, 29]; Lmnb2 [30]; Fmnl3 [31]) were found among the top hits with depleted sgRNAs in metastasis compared with tumors, underscoring the reliability of our screen.

From the top candidates identified in this screen, Polr1a was selected for further validation as a gene with well-characterized cellular function but with an underappreciated role in driving metastasis in melanoma and other cancers. RNA polymerase I (Polr1) is responsible for the transcription of 47S rDNA, resulting in the generation of 5.8S, 18S, and 28S rRNA needed for ribosome formation. Polr1 consists of several major subunits, including Po1r1a, and together with transcription factor RRN3, upstream binding factor (UBF), and SL-1 complex form the preinitiation complex at the rDNA [22]. Other polymerases involved in ribosome biogenesis are RNA polymerase II, responsible for ribosomal proteins’ genes’ transcription, and RNA polymerase III, which transcribes the 5S rRNA gene. Dysregulated ribosome biogenesis and hyperactivated RNA Polr1 activity have been associated with tumorigenesis and poor prognosis in patients [32]. We have shown that Polr1a did not influence cell viability or proliferation of melanoma cells; however, its downregulation inhibits cell migration and invasion. Therefore, the effect of Polr1a on the motility and invasiveness of melanoma cells likely contributes to its role in metastasis.

Although downregulation of Polr1a inhibited transcription of ribosomal RNA, to our surprise, it did not affect either steady-state levels of ribosomal RNA or global translation. However, Ribo-seq data reveal that Polr1a knockdown inhibited translation of a subset of mRNAs whose protein products may contribute to the observed effects of Polr1a on pro-metastatic properties of melanoma cells. One possible explanation of this phenomenon is that Polr1a, as a major Polr1 subunit, may cause a shift in ribosome heterogeneity that affects only a subset of translating mRNAs. A potential source of ribosome heterogeneity contributing to the difference in their functions and carcinogenesis can be in differential modifications of pre-rRNA nucleotides, such as pseudouridylation and 2ʹ-O-ribose methylation [33]. It is plausible that modulation of Polr1a function can disproportionally affect co-transcriptional modifications of rRNA, resulting in the biogenesis of ribosomes with specifically altered translation. RNA-polymerase I (Pol I) transcribes the rDNA loci within the nucleolus as individual transcriptional units, with multiple loci undergoing active transcription. The varying expression of distinct variant alleles contributes to the diversity observed in the population of cellular rRNAs [34]. If distinct rDNA loci code for rRNAs with varying specificity toward translating different mRNAs, then another possibility is that Polr1a downregulation could potentially result in the differential regulation of transcription of specific rRNA genes. Altogether, these changes in Polr1a machinery could potentially lead to the formation of ribosomes translating preferentially sets of genes important for the early stages of metastasis. Intriguing mechanism(s) of selective translational control by Polr1a will be the subject of a separate study.

Our Ribo-seq data identified the non-canonical NF-κB signaling pathway as one of the most strongly affected by Polr1a downregulation. NF-κB transcription factors can be activated in two ways: through canonical and non-canonical signaling. While canonical NF-κB is activated by ligands of a wide range of immune receptors and causes a rapid response, the non-canonical NF-κB pathway is characterized by a slower mode of action and more specific receptors [35]. Receptors, activating non-canonical NF-κB, mostly belong to the TNFR superfamily members: receptor activator for nuclear factor κB (RANK), lymphotoxin β-receptor (LTβR), CD40, B-cell-activating factor belonging to TNF family receptor (BAFFR), toll-like receptor 4 (TLR4) etc. Activation of these receptors induces nuclear factor-κB (NF-κB) inducing kinase (NIK), which in turn results in IKKα-dependent phosphorylation of p100 and its βTrCP-dependent ubiquitination and processing into p52. Processed p52, as a heterodimer with RelB, translocates into the nucleus to activate transcription activity [36]. Our data suggest that the suppression of melanoma metastatic properties that we observed after Polr1a downregulation is caused by the non-canonical NF-κB axis and its potential regulation of transcriptional activity of genes responsible for migration and invasion. While the role of non-canonical NF-κB signaling has been reported in driving multiple oncogenic processes, it is challenging to target it directly, while targeting Polr1a provides an alternative for downregulating non-canonical NF-κB signaling for therapeutic applications. Further studies are needed to elucidate the exact mechanisms of how ribosomal biogenesis and specifically Polr1a regulate the activity of non-canonical NF-κB signaling.

Melanoma metastasis involves homing to specific organs through lymphatic flow and chemotactic signals, directing cells to sites like lymph nodes, lungs, or liver [37]. Colonization requires genetic adaptations acquired at metastatic sites, enabling survival, immune evasion, and microenvironmental niche establishment [38]. This process faces high attrition rates, with <0.02% of circulating cells forming metastases due to barriers like tissue infiltration and immune responses [39]. Our findings show that Polr1a KD decreases the homing efficiency and colonization ability of melanoma cells. This may result from the Polr1a effects on migration and invasion properties, but there might also be other underlying mechanisms that are the subject of further investigation.

Elevated ribosomal biogenesis has been previously defined as a hallmark of cancer [33] and has been used as a therapeutic target for tumor growth inhibition. It was shown that pharmacological inhibition of Polr1a with BMH-21 or CX-5461 can inhibit the tumor growth of a variety of malignancies [17, 20, 23, 40]. Moreover, CX-5461 has already passed phase one of clinical trials as a drug for treating advanced hematological malignancies with both wild-type and mutant p53 [24], which creates a smooth avenue for the transition from preclinical to clinical trials of CX-5461 as a drug for melanoma metastasis treatment. One of the main challenges of targeted therapy remains the occurrence of resistance. For CX-5461, due to its effect on ribosome biogenesis, the therapeutic window is expected to be narrow, and it is expected to be used in combination with other drugs, such as MEK/ERK inhibitors and anti-PD-1 or anti-PD-1/CTLA-4 immunotherapy. Using the combinational therapy may help in preventing the development of resistance to Polr1 inhibition.

Overall, our results demonstrate that Polr1a is a potent regulator of melanoma metastasis and provide a foundation for a new potential utilization of POLR1 inhibitors as neoadjuvant therapy for the treatment of metastatic melanoma disease.

## Supplementary information

Fig. S1

Fig. S2

Fig. S3

Fig. S4

Fig. S5

Fig. S6

Supplemental Fig. legends

Supplemental table 1

Supplemental table 2

Supplemental tables and methods

## Data Availability

Raw RNA-seq and RIBO-seq data, as well as CRISPR library data generated during this study, are available at GEO (GSE302801, GSE302802 and GSE314397). The data generated in this study are available within the article and its supplementary data files. The rest of the raw data that support the findings of this study are available from the corresponding author on reasonable request.
