# Supplementary figures and images for: In vivo CRISPR knockout screen identifies Polr1a as a key driver and a potential therapeutic target for melanoma metastasis
Source: Oncogene. 2026 Jun 17;45(29):2978–87. doi: 10.1038/s41388-026-03851-4 (PMC13364679; doi:10.1038/s41388-026-03851-4)

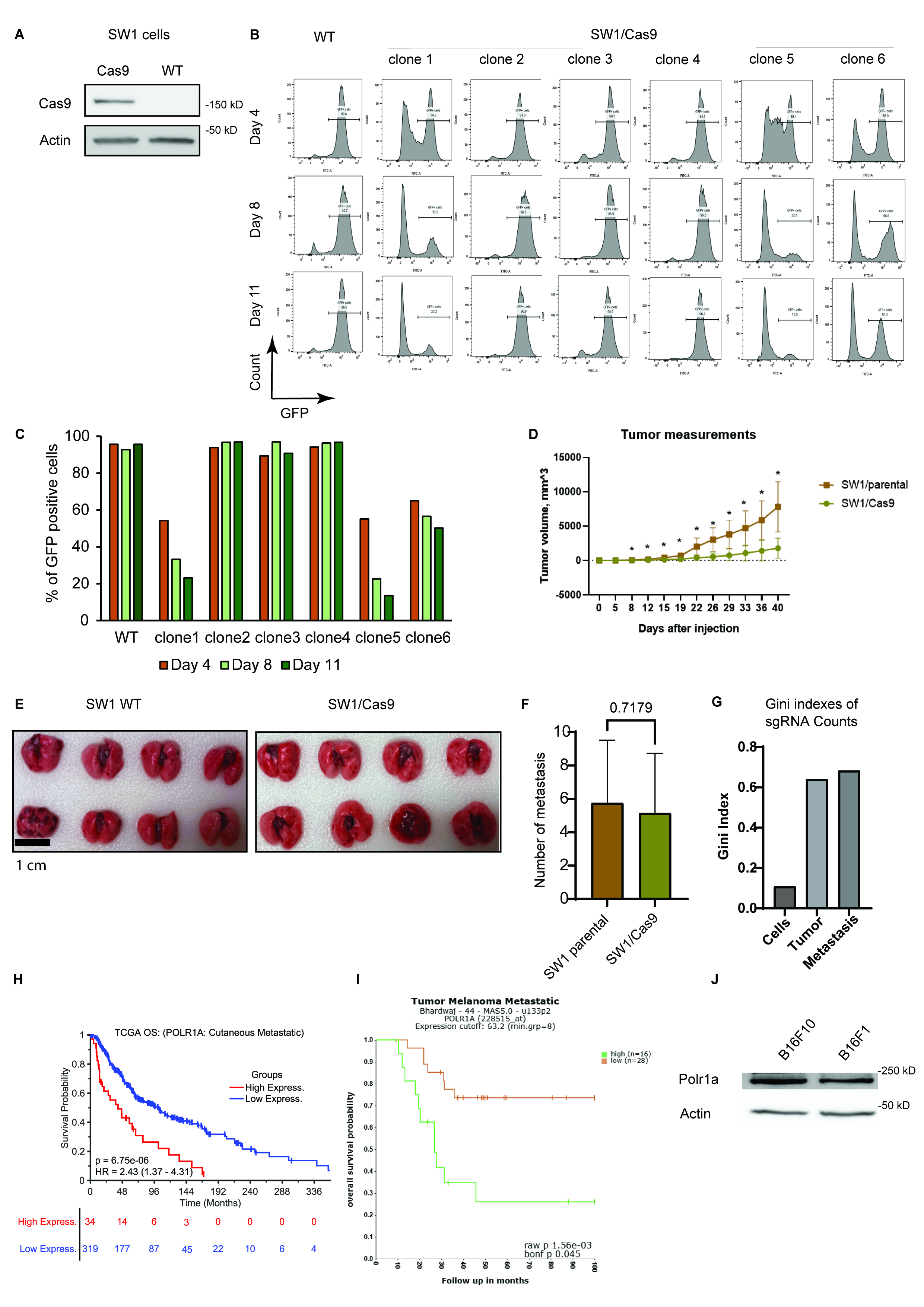

Supplement: Supplementary file 1 — Fig. S1 [file 41388_2026_3851_MOESM1_ESM.tif]

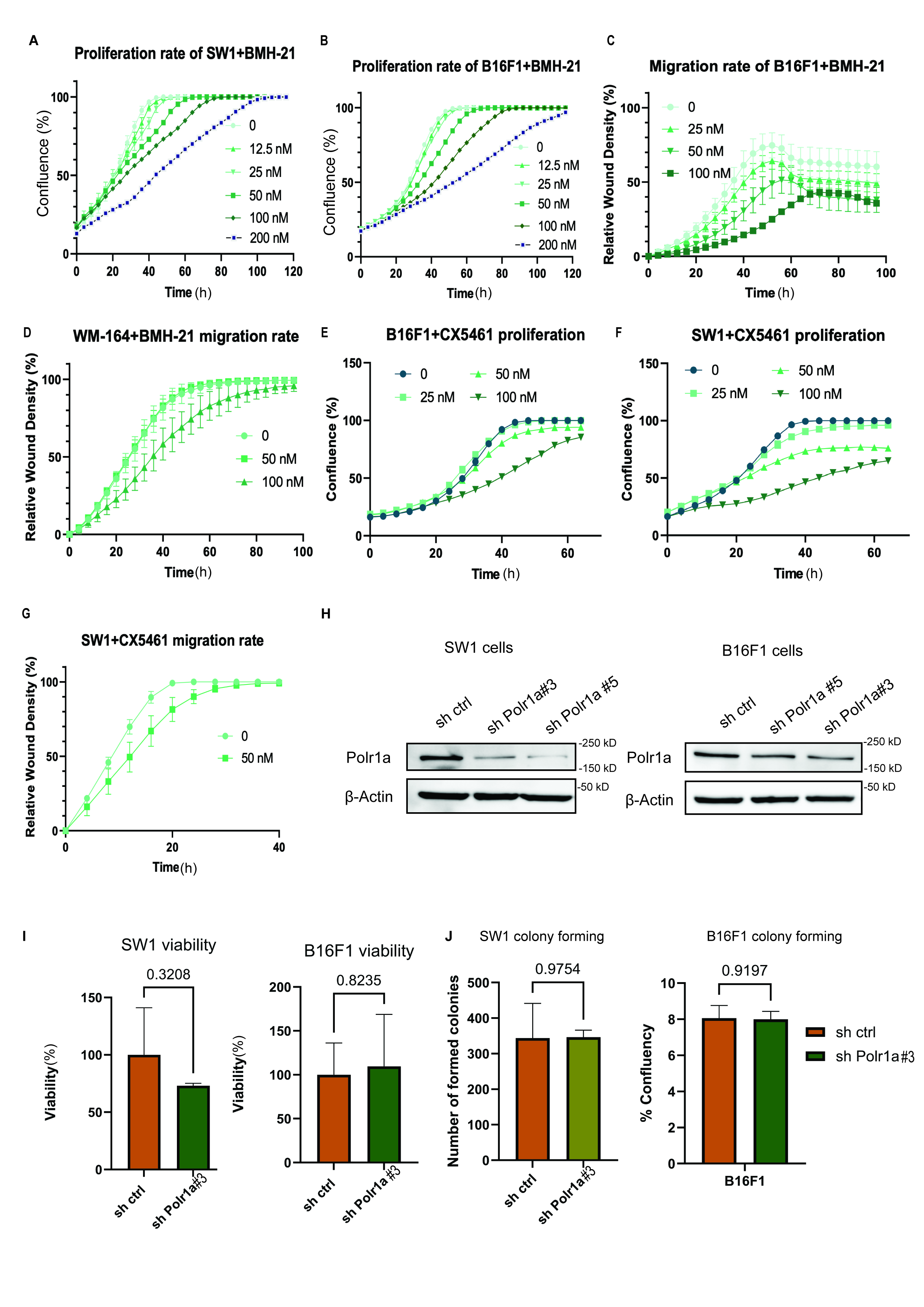

Supplement: Supplementary file 2 — Fig. S2 [file 41388_2026_3851_MOESM2_ESM.tif]

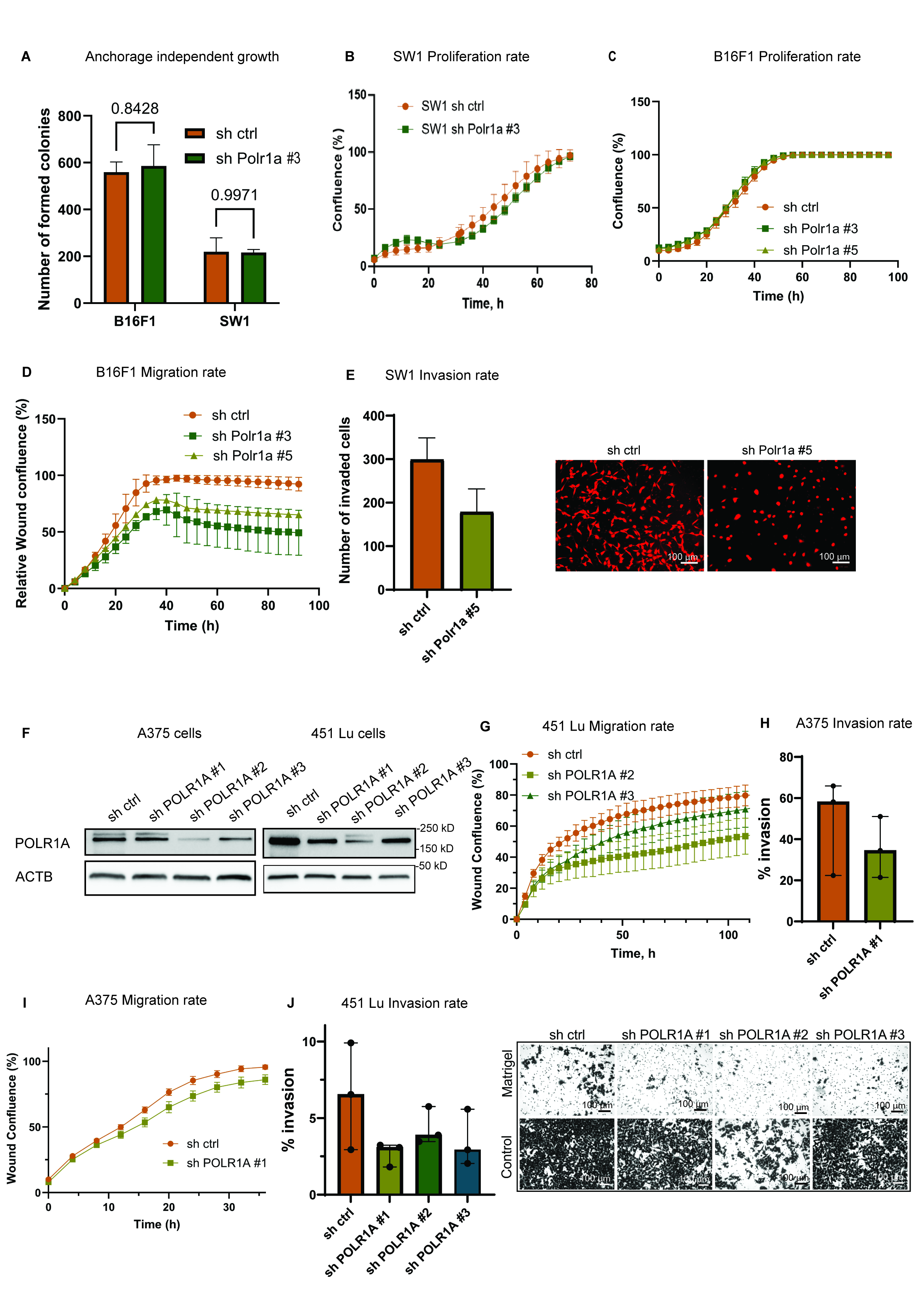

Supplement: Supplementary file 3 — Fig. S3 [file 41388_2026_3851_MOESM3_ESM.tif]

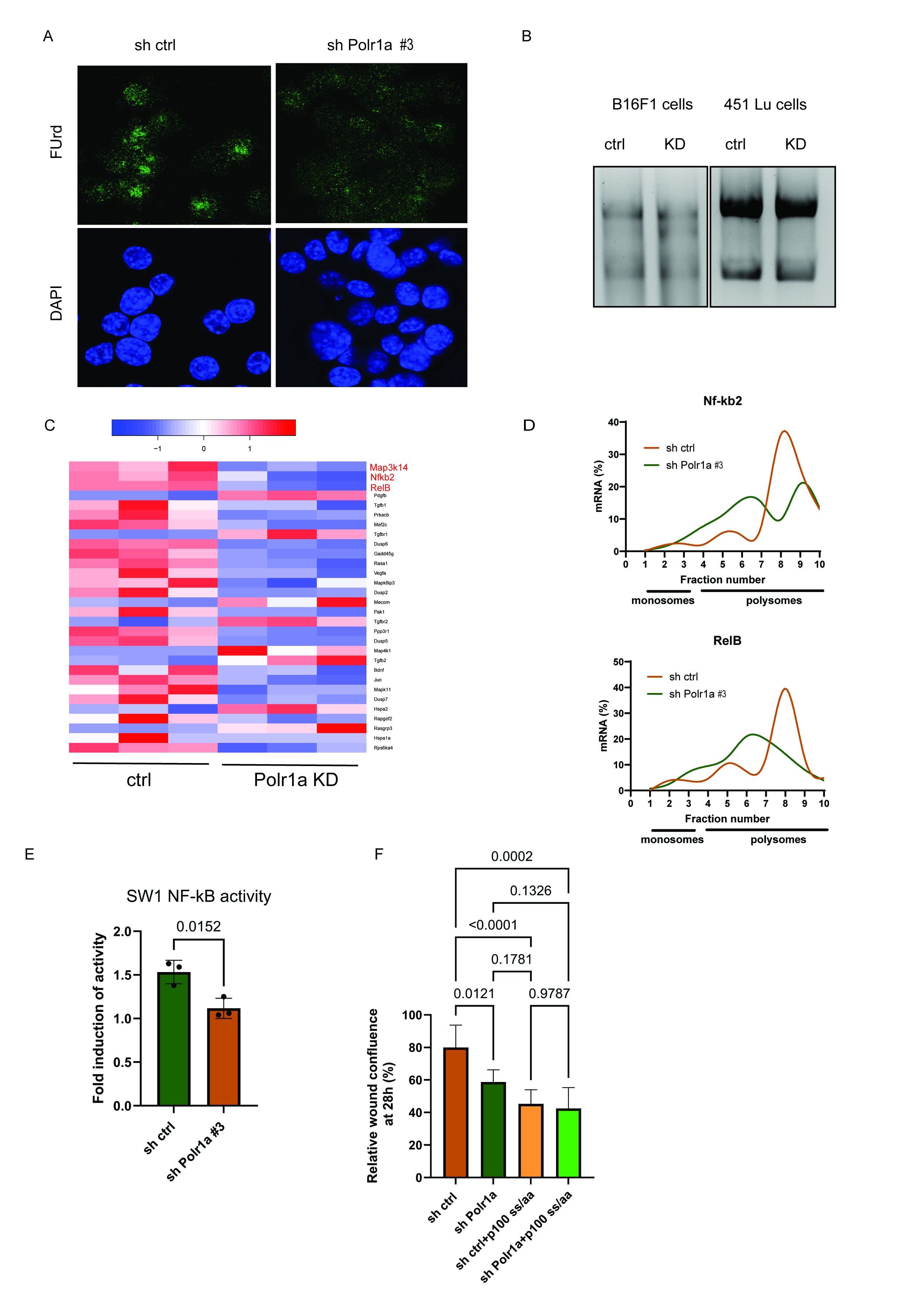

Supplement: Supplementary file 4 — Fig. S4 [file 41388_2026_3851_MOESM4_ESM.tif]

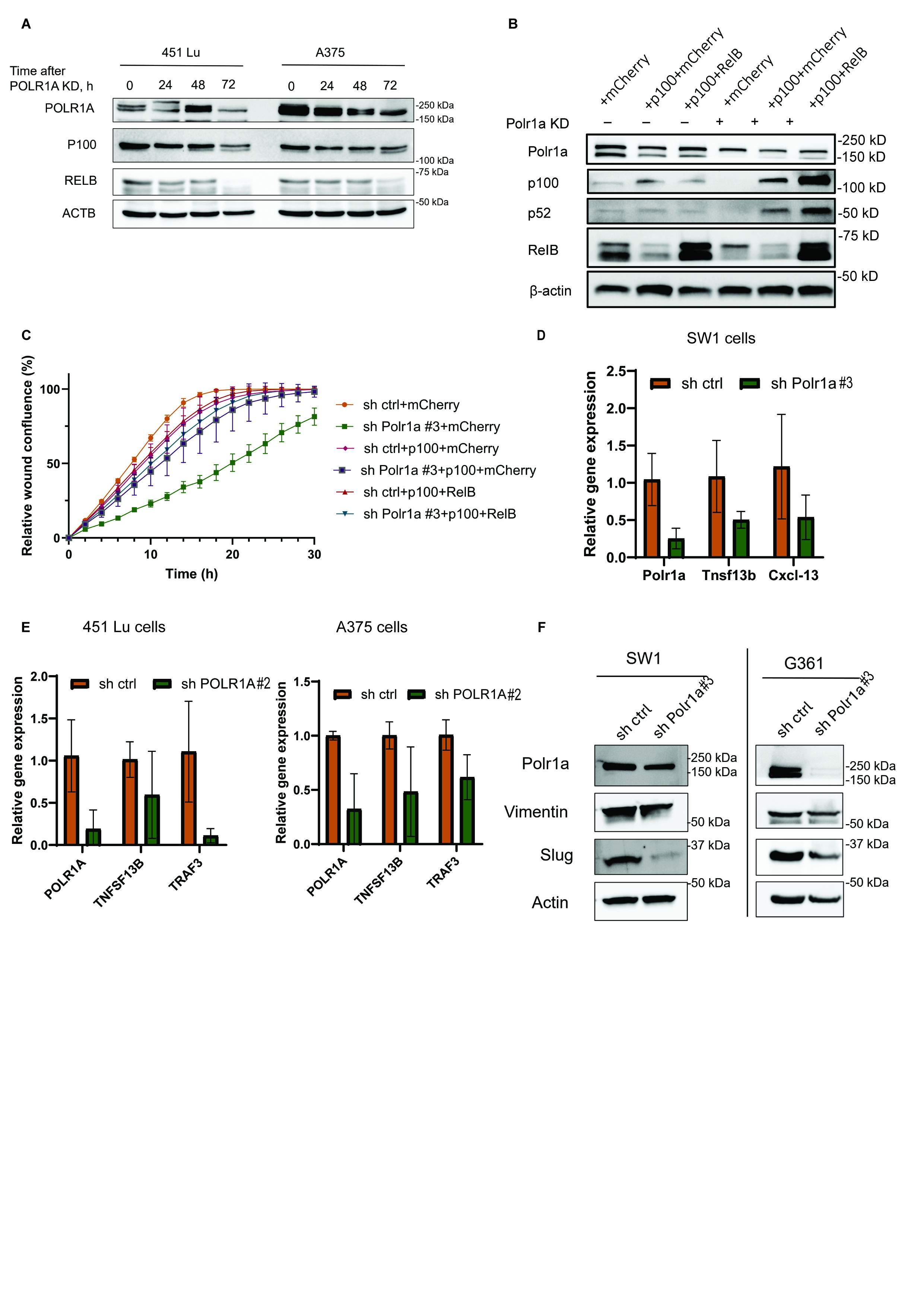

Supplement: Supplementary file 5 — Fig. S5 [file 41388_2026_3851_MOESM5_ESM.tif]

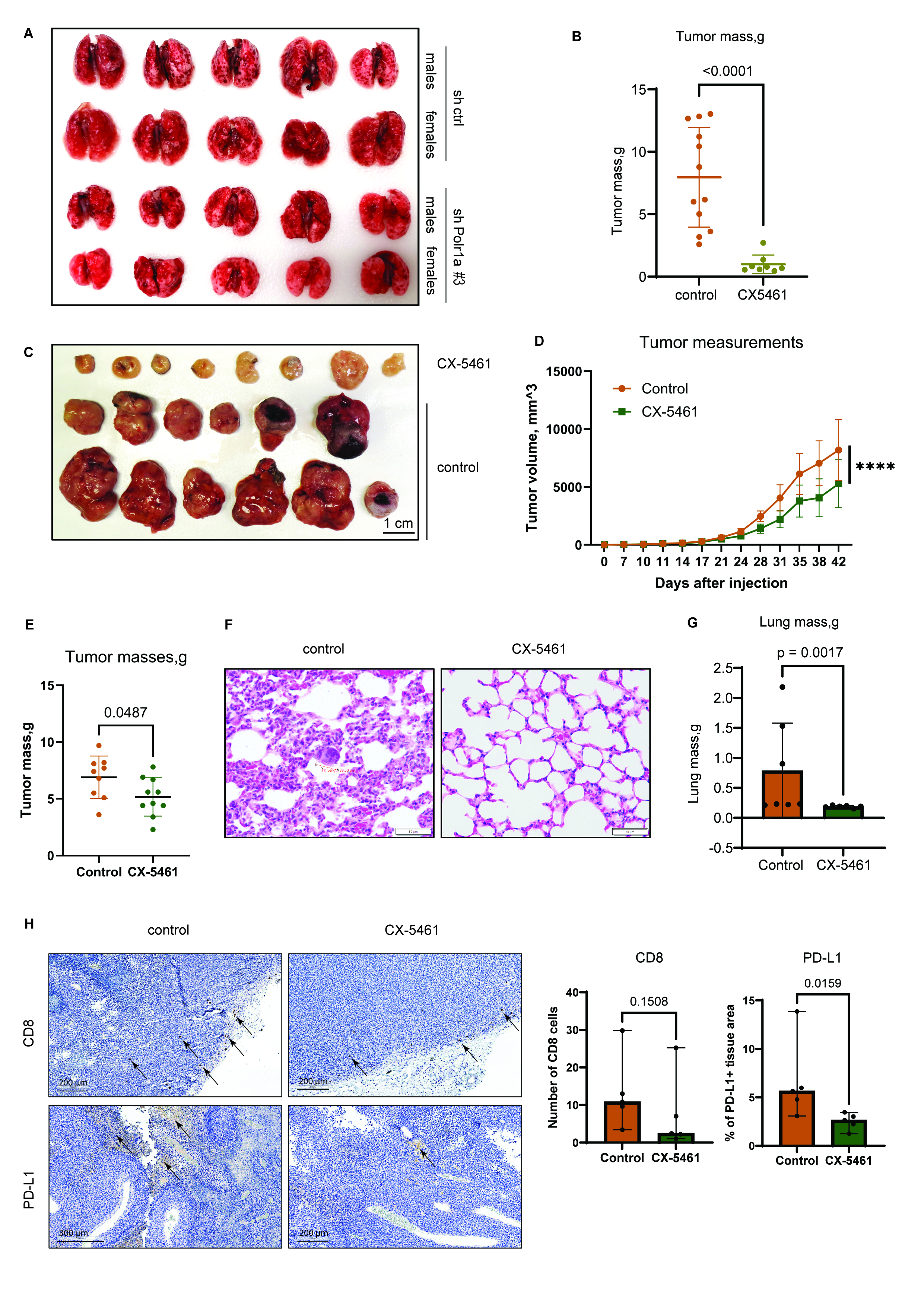

Supplement: Supplementary file 6 — Fig. S6 [file 41388_2026_3851_MOESM6_ESM.tif]
