# Supplementary material for: In vivo CRISPR knockout screen identifies Polr1a as a key driver and a potential therapeutic target for melanoma metastasis
Source: Oncogene. 2026 Jun 17;45(29):2978–87. doi: 10.1038/s41388-026-03851-4 (PMC13364679; doi:10.1038/s41388-026-03851-4)
Supplement: Supplementary file 7 — Supplemental Fig. legends [file 41388_2026_3851_MOESM7_ESM.docx]

**Supplemental figure legends**

Figure S1. Preparation for CRISPR knockout screen.

(A) Generation of stable SW1/Cas9 cell line: Cas9 expression detected with western blot. (B) GFP/Cas9 assay to identify the SW1/Cas9 clone with the highest Cas9 activity: GFP staining acquired with flow cytometry. (C) Statistics of GFP-positive SW1/Cas9 cells. In vivo testing of tumorigenicity of generated SW1/Cas9 clonal cell line: (D) Tumor growth dynamics in C3H mice bearing SW1/Cas9 subcutaneous tumors in comparison with WT SW1 cells (n=8/group, multiple unpaired t-tests). (E) Pictures of lungs with metastasis on day 40 (F) Number of macro metastasis (Mann-Whitney test, n=8/group). (G) Gini Indexes of CRISPR library samples (cells, tumor, and metastasis). Impact of POLR1A on overall survival of patients with (H) skin cutaneous melanoma: subset of metastatic melanoma patients from figure 1C (Kaplan Meier, n=353) or (I) metastatic melanoma (Kaplan Meier, n=44). (J) Polr1a content in the non-metastatic (B16F1) versus metastatic (B16F10) melanoma cell line.

Figure S2. Polr1a KD does not influence cell viability, colony-forming ability, and anchorage-independent growth.

(A) SW1 cells proliferation measured with IncuCyte live cell analysis imaging system after 24h pretreatment with corresponding doses of BMH-21. (B) B16F1 cells proliferation and (C) B16F1 cells migration rate measured with IncuCyte live cell analysis imaging system after 24h pretreatment with corresponding doses of BMH-21. (D) WM-164 cells' migration rate measured with IncuCyte live cell analysis imaging system after 24h pretreatment with corresponding doses of BMH-21. (E) B16F1 cells' proliferation measured with the IncuCyte live cell analysis imaging system after 24h pretreatment with corresponding doses of CX-5461. (F) SW1 cells proliferation and (G) SW1 cells migration rate measured with IncuCyte live cell analysis imaging system after 24h pretreatment with corresponding doses of CX-5461. (H) Western blot showing representative protein levels of Polr1a and actin in Polr1a KD and control KD in SW1 and B16F1 cells. (I) Viability of SW1 and B16F1 cells with Polr1a constitutive KD (XTT assay) estimated 24h after seeding cells (Unpaired t test, n=3/group, SW1: t=1.132, df=4; B16F1:t=0.2381, df=4 ). (J) Influence of Polr1a KD on colony forming of SW1 (Paired t test, n= 3, t=0.03481, df=2) and B16F1 cells (Paired t test, n= 3/group, t=0.1140, df=2). (B)

Figure S3. Polr1a KD does not influence proliferation, but inhibits cell migration and invasion.

(A) Anchorage-independent growth of SW1 and B16F1 cells with Polr1a KD (Two-way ANOVA, n=3/group). Proliferation of SW1 (B) and B16F1 (C) cells measured with IncuCyte live cell analysis. (D) B16F1 cells migration rate measured with IncuCyte live cell analysis imaging system. (E) Quantification of invasion of SW1 cells with Polr1a KD #5 (Mann-Whitney test, n=4/group, N=3) and representative pictures of cells invaded through the matrigel. (F) Western blot showing representative protein levels of POLR1A and ACTIN in POLR1A KD and control KD A375 and 451 Lu cells after 2 days of induction with 1 ug/mL of doxycycline. (G) 451Lu cells migration rate measured with IncuCyte live cell analysis imaging system. (H) Quantification of invasion rate of A375 cells (Unpaired t-test, n=3/group). (I) A375 cells migration rate measured with IncuCyte live cell analysis imaging system. (J) Quantification of invasion rate of 451Lu cells and representative pictures (Unpaired t-test, n=3/group).

Figure S4. Genes regulated by Polr1a on the transcriptional and translational levels.

(A) Immunofluorescent staining for rRNA synthesis (FUrd) in Polr1a KD and control KD SW1 cells. (B) 28s and 18s rRNA in control and Polr1a KD cells (1% agarose gel). (C) Heatmap of genes involved in MAPK/NF-κB pathway affected by Polr1a KD on the translational level. 3 columns represent 3 biological replicates. (D) Polysome profiling for NF-kB2 and RelB mRNAs of SW1 Polr1a KD cells. (E) NF-kB reporter assay in SW1 cells (Unpaired t test, t=4.068, df=4; n=3). (F) The summary of the ordinary one-way ANOVA at 28 hours of B16F1 cells migration measured with IncuCyte live cell analysis imaging system (n=4-7).

Figure S5. Mechanism of action.

(A) Western blot showing the levels of p100 and RelB in the 451 Lu and A375 cells after the doxycycline induction of POLR1A KD. (B) Representative western blot showing the levels of p100, p52, and RelB in the cells used for migration assay in (C). (C) SW1 cells with re-expressed p100 and RelB migration rate measured with IncuCyte live cell analysis imaging system (same experiment as Figure 3G, but with combination of p100 and RelB). (D&E) qRT-PCR of genes targets of the non-canonical NF-κB pathway in SW1 cells, A375 and 451 Lu cells (Unpaired t test with Welch correction, n=3). (F) Western blot showing the levels of EMT markers Vimentin and Slug in SW1 and G361 cells with Polr1a KD.

Figure S6. Polr1a inhibition as a therapeutic strategy.

(A) Pictures of lungs with metastasis on day 14 after IV injection. (B) Tumor masses of mice treated with CX-5461 on day 37, g (n ≥ 8 per group). Data are represented as mean ±SD. P-values are comparing the control group vs the CX-5461-treated group by the Mann-Whitney test. (C) Pictures of tumors of C3H mice treated with CX-5461 (D) Tumor growth dynamics in NSG mice treated with CX-5461 (n ≥ 9 per group). Data are represented as mean ±SD. p values are comparing the control group vs CX-5461 treated group by unpaired t test with Welch correction. (E) Tumor masses of mice treated with CX-5461 on day 42, g (n ≥ 9 per group). Data are represented as mean ±SD. P-values are comparing the control group vs the CX-5461-treated group by the unpaired t test. (F) H&E-stained lungs of NSG mice treated with CX-5461 or vehicle. (G) Mass of lungs of the mice that underwent surgical excision of SW1 tumors and were treated with CX-5461 (Mann-Whitney test, n=7). (H) CD8 and PD-L1 IHC staining of SW1 tumors of mice treated with CX-5461. Representative pictures (left panel) and quantification (right panel).
