# Supplementary material for: In vivo CRISPR knockout screen identifies Polr1a as a key driver and a potential therapeutic target for melanoma metastasis
Source: Oncogene. 2026 Jun 17;45(29):2978–87. doi: 10.1038/s41388-026-03851-4 (PMC13364679; doi:10.1038/s41388-026-03851-4)
Supplement: Supplementary file 10 — Supplemental tables and methods [file 41388_2026_3851_MOESM10_ESM.docx]

**Supplemental tables**

**Table S3.** Plasmids used in the study

| Construct/Plasmid Name | Company | Catalog # |
| --- | --- | --- |
| pXPR-011 | Addgene | #59702 |
| lentiCas9-Blast | Addgene | #52962 |
| pLentiGuide | Addgene | #117986 |
| SMARTvector Inducible Human POLR1A shRNA #1 | Dharmacon Horizon | GSGM11941-248141446  (Target sequence:  TACCGTCCCGTCAATCGCCT) |
| SMARTvector Inducible Human POLR1A shRNA #2 | Dharmacon Horizon | GSGM11941-248554432  (Target sequence:  GTGAAAGCTTTGAACCTAAA) |
| SMARTvector Inducible Human POLR1A shRNA #3 | Dharmacon Horizon | GSGM11941-248032730  (Target sequence:  GCTTGAGCGAATTCTCAGCA) |
| SMARTvector Inducible Non-targeting Control | Dharmacon Horizon | VSC6570 |
| shPolr1a N3 const (m) | Sigma Aldrich | TRCN0000288221 (target sequence: CCTTCAGCAAATGGCCTGTAT) |
| shPolr1a N5 const (m) | Sigma Aldrich | TRCN0000298426 (target sequence:  CGGAATAAGTTCCAGGTGTAT) |
| MISSION® TRC2 pLKO.5-puro Non-Mammalian shRNA Control | Sigma Aldrich | SHC202 |
| pCLXSN(GFP)-hp100 SS/AA | Addgene | #174735 |
| pCLXSN(GFP)-hp100 | Addgene | #174734 |
| pFUW-tetO-Relb | Addgene | #139810 |
| pFUW-tetO-mCherry | Addgene | #193060 |
| GFP-RPA194 | Addgene | #17660 |
| pcDNA3-EGFP | Addgene | #13031 |
| psPAX2 | Addgene | #12260 |
| pMD2.G | Addgene | #12259 |

**Table S4.** Primers used in this study.

| Gene/primer name | Fwd | Rev |
| --- | --- | --- |
| NGS-Lib -1 | AATGATACGGCGACCACCGAGATCTA CACTCTTTCCCTACACGACGCTCTTCC  GATCTTAAGTAGAGGCTTTATATATCT TGTGGAAAGGACGAAACACC | CAAGCAGAAGACGGCATACGAGATTC GCCTT GGTGACTGGAGTTCAGACGTG  TGCTCTTCCGATCTCCGACTCGGTGCC ACTTTTTCAA |
| NGS-Lib -2 | AATGATACGGCGACCACCGAGATCTA CACTCTTTCCCTACACGACGCTCTTCC  GATCTATCATGCTTAGCTTTATATATC TTGTGGAAAGGACGAAACACC | CAAGCAGAAGACGGCATACGAGATAT AGCGTC GTGACTGGAGTTCAGACGTG  TGCTCTTCCGATCTCCGACTCGGTGCC ACTTTTTCAA |
| NGS-Lib -3 | AATGATACGGCGACCACCGAGATCTA CACTCTTTCCCTACACGACGCTCTTCC  GATCTGATGCACATCTGCTTTATATAT CTTGTGGAAAGGACGAAACACC | CAAGCAGAAGACGGCATACGAGATGA AGAA GTGTGACTGGAGTTCAGACGTG  TGCTCTTCCGATCTCCGACTCGGTGCC ACTTTTTCAA |
| NGS-Lib -4 | AATGATACGGCGACCACCGAGATCTA CACTCTTTCCCTACACGACGCTCTTCC  GATCTCGATTGCTCGACGCTTTATATA TCTTGTGGAAAGGACGAAACACC | CAAGCAGAAGACGGCATACGAGATAT TCTA GGGTGACTGGAGTTCAGACGTG  TGCTCTTCCGATCTCCGACTCGGTGCC ACTTTTTCAA |
| Luciferase | CCCATCTTCGGCAACCAGAT | GTACATGAGCACGACCCGAA |
| Rps-18 (m) | CCGCCGCCATGTCTCTAGT | CCCTCTTGGTGAGGTCGATG |
| TNFSF13B | CCGCGGGACTGAAAATCT | CTTCTAGGGCACTTCCCCTTT |
| TRAF3 | ACTGCAAGAGTCAGGTTCCG | GGCTGTCTATCACTCGCTGT |
| RPS18 (h) | GGCGGCGGAAAATAGCCTTT | ATCACACGTTCCACCTCATCCT |
| Tnsf13b(m) | TGTTCCATGGCTTCTCAGCTT | GGTCCGTGTATAGAACCTGGC |
| Cxcl-13(m) | ATTCAAGTTACGCCCCCTGG | TTGGCACGAGGATTCACACA |
| Polr1a (m) | GCGGCTGACTGGAACTTCTC | CCCAGGTAGTCCACGTATCG |

**Table S5.** Antibodies

| Gene Name | Company | Catalog # | Application | Dilution |
| --- | --- | --- | --- | --- |
| Polr1a | Cell Signaling Technologies | #24799 | WB | 1:1000 |
| beta-Actin | Cell Signaling Technologies | 4970 | WB | 1:4000 |
| HRP-linked anti-rabbit | Cell Signaling Technologies | 7074 | WB | 1:2000 |
| Monoclonal Anti-BrdU antibody produced in mouse | Sigma | B2531-100UL | IF | 1:50 |
| NF-κB2 p100/p52 | Cell Signaling Technologies | 4882T | WB | 1:1000 |
| RelB (C1E4) Rabbit mAb | Cell Signaling Technologies | 4922T | WB | 1:1000 |
| RPA194 Antibody (C-1) | Santa Cruz Santa Cruz | sc-48385 | IHC | 1:100 |
| Alexa488 secondary anti-mouse antibodies | Jackson Immuno Research | 715-545-151 | IF | 1:400 |
| Vimentin | Cell Signaling Technologies | 49398T | WB | 1:1000 |
| Slug | Cell Signaling Technologies | 49398T | WB | 1:1000 |
| CD8α (D4W2Z) XP® Rabbit mAb | Cell Signaling Technologies | 98941T | IHC | 1:200 |
| PD-L1 (D5V3B) Rabbit mAb | Cell Signaling Technologies | 64988S | IHC | 1:100 |

**Table S6.** Body masses of vehicle-treated control mice

| **Days after tumor initiation** | **Day 17** | **Day 21** | **Day 24** | **Day 31** | **Day 38** | **Day 45** | **Day 51** |
| --- | --- | --- | --- | --- | --- | --- | --- |
| Ear mark |  |  |  |  |  |  |  |
| No |  | 27 | 26 | 26 | 28 | 29 | 29 |
| R | 24 | 26 | 25 | 27 | 28 | 27 | - |
| L | 24 | 25 | 23 | 24 | 24 | 25 | 26 |
| RR | 24 | 24 | 25 | 26 | 27 | 22 | - |
| LL | 24 | 25 | 26 | 26 | 27 | 27 | - |
| RR | 22 | 20 | 22 | 23 | 24 | 25 | - |
| LL | 21 | 22 | 21 | 20 | 23 | 23 | - |
| **Mean** | **23.17** | **23.67** | **23.67** | **24.33** | **25.50** | **24.83** | **26.00** |
| **SD** | **1.33** | **2.41** | **2.00** | **2.44** | **2.12** | **2.44** | **2.12** |

**Table S7.** Body masses of mice treated with CX-5461

| **Days after tumor initiation** | **Day 17** | **Day 21** | **Day 24** | **Day 31** | **Day 38** | **Day 45** | **Day 51** |
| --- | --- | --- | --- | --- | --- | --- | --- |
| Ear mark |  |  |  |  |  |  |  |
| LL |  | 23 | 23 | 24 | 25 | 24 |  |
| No | 25 | 24 | 25 | 26 | 26 | 27 | 27 |
| L | 23 | 24 | 25 | 27 | 27 | 27 | - |
| L | 23 | 22 | 22 | 23 | 22 | 20 | - |
| RR | 19 | 18 | 19 | 20 | 20 | 21 | 20 |
| L | 20 | 19 | 20 | 20 | 20 | 18 | - |
| **Mean** | **22.00** | **21.67** | **22.33** | **23.33** | **23.33** | **22.83** | **23.50** |
| **SD** | **2.45** | **2.58** | **2.50** | **2.94** | **3.08** | **3.76** | **4.95** |

**Supplemental materials and methods**

### Cell culture

Human embryonic kidney T-large antigen transformed cells (HEK293T), B16F1, B16F10, A375 cells were obtained from ATCC. Mouse melanoma cell line SW1, derived from K-1735p lung metastasis, was a generous gift from Dr. Ze’ev Ronai of Sanford Burnham Prebys Medical Discovery Institute (1). The 451 Lu cell line was obtained from Rockland Immunochemicals, Inc. (Rockland). Cells were maintained in DMEM (Corning) supplemented with 10% FBS (FBS, Gibco by Life Technologies, Carlsbad, CA, USA) and 1% penicillin-streptomycin (Corning, NY, USA) in the humidified incubator at 37°C, 5% CO_2._ Cells were routinely checked for mycoplasma with MycoAlert PLUS Mycoplasma Detection Kit (Lonza, Walkersville, MD). Cell lines' authenticity testing was performed at Labcorp, and STR profiles were confirmed using Cellosaurus references.

### Stable cell line generation by lentiviral transduction

LentiCas9-Blast was a gift from Feng Zhang (Addgene plasmid # 52962; http://n2t.net/addgene:52962 ; RRID:Addgene_52962). pCLXSN(GFP)-hp100 SS/AA was a gift from Shao-Cong Sun (Addgene plasmid # 174735 ; http://n2t.net/addgene:174735 ; RRID:Addgene_174735). pCLXSN(GFP)-hp100 was a gift from Shao-Cong Sun (Addgene plasmid # 174734 ; http://n2t.net/addgene:174734 ; RRID:Addgene_174734). pFUW-tetO-Relb was a gift from Filipe Pereira (Addgene plasmid # 139810 ; http://n2t.net/addgene:139810 ; RRID:Addgene_139810). pFUW-tetO-mCherry was a gift from Filipe Pereira (Addgene plasmid # 193060 ; http://n2t.net/addgene:193060 ; RRID:Addgene_193060). GFP-RPA194 was a gift from Tom Misteli (Addgene plasmid # 17660 ; http://n2t.net/addgene:17660 ; RRID:Addgene_17660). pcDNA3-EGFP was a gift from Doug Golenbock (Addgene plasmid # 13031 ; http://n2t.net/addgene:13031 ; RRID:Addgene_13031). psPAX2 was a gift from Didier Trono (Addgene plasmid # 12260 ; http://n2t.net/addgene:12260 ; RRID:Addgene_12260). pMD2.G was a gift from Didier Trono (Addgene plasmid # 12259 ; http://n2t.net/addgene:12259 ; RRID:Addgene_12259). Lentiviral vectors with short hairpin RNA (shRNA) against Polr1a or non-targeting control with constitutive expression were purchased from Sigma (St. Louis, MO), shRNAs with doxycycline-inducible expression were purchased from Dharmacon Horizon. Information about plasmids is provided in the Supplementary Materials and Methods, **Table S3**. Virus production and transfections were carried out as previously described (2). Briefly, HEK293T cells were transfected using a calcium-phosphate method with 10 μg of transfer plasmid, 10 μg of psPAX2 and 7.5 μg of pMD2G. The virus was collected and concentrated after 72 h. Target cells were transduced with the virus in the presence of 8 μg/mL polybrene and selected on 1 μg/mL of puromycin until all parental control cells were dead. Most in vitro experiments were performed with at least 2 different shRNA constructs. Representative results obtained with one of the constructs are shown in the figures.

### Survival analysis

To evaluate the clinical relevance of **POLR1A** expression in melanoma, we analyzed publicly available datasets using multiple tools. First, we examined TCGA melanoma data (n = 459) using the survminer R package (<https://github.com/kassambara/survminer>). Kaplan-Meier curves were used to visualize survival differences between high and low Polr1a expression groups, and **log-rank tests** were used to calculate p-values.

To further explore the association between Polr1a expression and overall survival, we utilized the **R2 Genomics Analysis and Visualization Platform** (<https://r2.amc.nl>). Kaplan-Meier analyses were performed on two additional datasets: a subset of TCGA Skin Cutaneous Melanoma tumors (n = 367)(3) and a metastatic melanoma cohort from Bhardwaj et al. (n = 44). The optimal expression cutoff was determined using the “scan” mode in R2.

### GFP Cas9 activity assay

pXPR_011 was a gift from John Doench & David Root (Addgene plasmid # 59702; http://n2t.net/addgene:59702; RRID: Addgene_59702). This plasmid carries both the GFP coding region and sgRNA targeting GFP. Thus, in cells with active Cas9, the GFP is cleaved and Cas9 activity can be estimated in reverse proportionality to the GFP fluorescence level decrease in comparison with parental cells transduced with the same pXPR-011 plasmid. SW1/Cas9 single-cell clones and SW1 parental cells were transduced with pXPR_011 at MOI=1 in the presence of 8 μg/mL polybrene. 48h after transduction 2 μg/mL of puromycin was added to select the cells. Samples of parental and SW1/Cas9 clonal cells were harvested after 4,8, and 11 days of selection and GFP fluorescence level was analyzed using BD FACS Canto flow cytometer.

### XTT assay

SW1, B16F1 cells (1×10^4^ /well) at the logarithmic phase of growth were plated into 96-well plates overnight. The next day CyQUANT™ XTT Cell Viability Assay (Invitrogen, X12223) was performed according to the manufacturer’s instructions to estimate cell viability. 70 μL of XTT and Electron Coupling Reagent mixture was added to each well and the plate was incubated for 4 hours at 37 °C, 5% CO_2_. After completion of incubation absorbance was measured at 450 nm and 660 nm using a CLARIOstar plate reader. Cell viability was calculated as the percentage of optical densities in wells with KD normalized to the optical density of sh ctrl cells (100%).

### Colony-forming assay

300 cells (SW1) or 500 cells (B16F1, B16F10) were seeded in 6-well plates. After 4 days cells were fixed in formalin, stained with 2% gentian violet in methanol (Ricca, #3235-4) for 30 minutes, washed in tap water, and dried at room temperature for 24 h. The number of colonies was counted under the microscope. Images were acquired using the ChemiDoc MP imaging system (Bio-Rad, Hercules, CA, USA), and colony confluency was quantified using ImageJ(4).

### Anchorage-independent growth

Soft agar colony formation assay was performed as previously described (5). Briefly, 1.5 mL of 1:1 mixture of 1% soft agar and complete media (DMEM with 10% FBS and 1% penicillin-streptomycin) were layered on the bottom of the 6-well plate and were allowed to solidify at room temperature. For the upper layer, 1.5 mL of 1:1 mixture of 0.6 % soft agar and cell suspension containing 1000 cells were pipetted on top of the bottom layer and were allowed to solidify at room temperature. Colonies were grown for 18 days, then 200 μL of nitroblue tetrazolium chloride solution per well was added and plates were incubated overnight at 37 °C. The number of colonies was counted under the microscope.

### Proliferation assay (IncuCyte)

SW1, B16F1 mouse melanoma cells, A375, 451 Lu human melanoma cells (1×10^4^) were seeded in a 96-well plate (Corning, 353075). Cells with doxycycline-inducible knockdown were pretreated with 1 μg/mL doxycycline for 48 hours and plated in growth media supplemented with doxycycline. Cells were allowed to attach for 2 hours in the humidified incubator, and then the plate was imaged and quantified using IncuCyte ZOOM imaging system using standard scan type. 3 images/well were collected every 4 hours until confluency reached 100%.

### Migration assay (IncuCyte)

SW1, B16F1 cells (8×10^4^), A375, 451 Lu cells (7×10^4^) were seeded in a 96-well ImageLock plate (Sartorius, 4379) overnight. Cells with doxycycline-inducible knockdown were pretreated with 1 μg/mL doxycycline for 24 hours and plated in growth media supplemented with doxycycline. The scratch was made the next day when cells reached confluency using the WoundMaker (Sartorius, 4563) following the manufacturer’s protocol. The plate was imaged and quantified using IncuCyte ZOOM imaging system using Scratch wound scan type. 2 images/well were collected every 4 hours until wound confluency reached 100%.

### Invasion assay

Invasion assay was performed using Corning Biocoat Matrigel invasion chambers (Corning 354480) and control inserts (Corning 354578) according to the manufacturer’s instructions. Briefly, cells with doxycycline-inducible knockdown were pretreated with 1 μg/mL doxycycline for 48 hours. 25×10^3^ (SW1,451 Lu cells), 4×10^4^ (A375 cells) were seeded in 500 μL of serum-free media (supplemented with 1 μg/mL doxycycline in case of A375 and 451 Lu) in the upper chambers. 750 μL of complete media with 10% FBS as a chemoattractant was added into the wells of the companion plates. Cells were allowed to invade/migrate for 26 hours (A375) or 48 hours (SW1, 451 Lu), then fixed for 5 minutes in 100% methanol and stained for 30 minutes with 0.1 % gentian violet in 5% methanol (Ricca, #3235-4), rinsed and dried. For each insert 5 random fields of view were selected for acquisition using Biorad ZOE Fluorescent Cell Imager in brightfield mode. The number of cells invaded through Matrigel or migrated in control inserts was quantified using ImageJ software (<https://imagej.nih.gov/ij/index.html>). The percentage of invasion was calculated according to the following equation: % invasion = area of invaded cells through Matrigel inserts/area of migrated cells through control inserts × 100.

### Western blot analysis

Western blotting was performed according to the standard protocol. Briefly, 50 μg of total protein was separated on 10% polyacrylamide/SDS gel and transferred to PVDF membranes, the membranes were blocked with 5% (w/v) milk in Tris-buffered saline with 0.1% Tween-20 (TBST). Incubation with primary antibodies was performed overnight at 4°C. Membranes were washed 3 times in TBST and incubated with secondary antibodies for 1 hour at RT. Staining was developed using WesternSure Premium Chemiluminescent substrate (Li-cor, D21121-12) and images were acquired using the ChemiDoc MP imaging system (Bio-Rad, Hercules, CA, USA). Information about antibodies is provided in the Supplementary Materials and Methods **Table S5**.

### Riboseq and RNA seq

For RNA seq analysis SW1 sh ctrl or sh Polr1a cells were collected and pellets were immediately frozen at -80 °C. For Ribo seq analysis SW1 sh ctrl or sh Polr1a cells were treated with 0.1 mg/mL of cycloheximide (Sigma Aldrich, C4859-1ML) for 2 min, then collected in ice-cold PBS with 0.1 mg/mL of cycloheximide, pelleted, snap-frozen in liquid nitrogen, and stored at -80. Further sample preparation and sequencing were done at CD Genomics (NY, USA).

During bioinformatic analysis genes were classified into five groups according to the expression changes at the transcriptional and translational levels: (I) Transcription (significantly different only at the transcriptional level); (II) Translation (significantly different only at translational level); (III) Homodirection (significantly different at both levels and have the same trends); (IV) Opposite (significantly different at both levels and have the opposite trends); (V) Unchanged (not significantly different at both levels). Genes of the above five groups were subjected to KEGG pathways analysis. KEGG enrichment bar charts: the graphs were generated using the pathways with the Q value<0.05. The ordinate is the pathway, and the abscissa is the percentage of the number of differential genes in the pathway divided by all the numbers in the pathway. The darker the color, the smaller the Q value. The value on the column is the number and Q value of the pathway. Translational efficiency (TE) was calculated as the ratio of translating mRNAs to the total mRNAs of a gene. It is an important indicator to describe the RNA translation process. RiboDiff (6) was used to identify differential TE genes across sample groups, and genes with a fold change≥2 and a false discovery rate (FDR) <0.05 in comparison were considered as significant differential TE genes (DTEGs).

### CX-5461 neoadjuvant treatment

0,7 × 10^6^ of SW1 cells were resuspended in phosphate-buffered saline (PBS, Corning) and injected subcutaneously to C3H/HeJ mice. When tumor size reached 120 mm^3^ (on average) mice were randomly assigned to the CX-5461 or vehicle (sodium-phosphate buffer, pH=4.6) treatment groups (n=>9) using RandoMice software. CX-5461 was injected intraperitoneally 50 mg/kg twice a week during the first two weeks and then once a week until the endpoint. Once the tumors reached 350-450 mm^3^, they were resected. Lungs were harvested on day 35 after the surgery or when mice reached the moribund state, whichever happened earlier. Macrometastases were counted, and lung mass was measured to characterize the lungs that developed enormous metastases. Lung tissues were fixed in 10% formalin and H&E stained. Tumor burden was calculated using the Fiji software(7) as a percentage of hematoxylin-stained area (metastasis) to the eosin-stained area (total lung tissue area).

Animals that developed locally relapsing tumors were excluded from the analysis.

### Tumor cells’ lung colonization assay

1× 10^6^ of SW1 single-cell clones of sh ctrl or sh Polr1a were injected IV to C3H/HeJ mice. Lungs were collected 14 days after the cell injection. Tissues were fixed in 10% formalin and H&E stained. Tumor burden was calculated using the Fiji software as a percentage of hematoxylin-stained area (metastasis) to the eosin-stained area (total lung tissue area).

### Circulating tumor cells’ evaluation

2× 10^6^ of SW1 single-cell clones of Luc/sh ctrl or shPolr1a cells were IV injected to C3H/HeJ mice. Blood was collected from the saphenous vein after 30 min;1.5h and 2.5h. 100 μL of blood from each probe was treated with RBC lysis buffer (Biolegend, 420301). DNA was isolated from the remaining cell pellet using a DNeasy Blood & Tissue Kit (69504) per the manufacturer’s instructions. 50 ng of DNA sample was amplified in a qPCR reaction with primers detecting the luciferase sequence introduced in the SW1 cells and not present in any blood cells. For quantification of results, the calibration curve was generated by mixing 1-25 × 10^3^ of SW1 Luc/sh ctrl or shPolr1a cells with intact blood and subjecting it to the DNA isolation and qPCR following the procedure described above. Data was normalized on the RPS18 signal using the standard ΔΔCq Calculation Method.

### FUrd assay

The assay was performed as previously described for semiquantitative analysis of Pol I transcription in living cells(8). Briefly, SW1 cells with constitutive Polra KD were plated on coverslips and grown until they reached 80 % confluence. Positive control samples were treated with 0.04 μg/mL of Actinomycin D (Invitrogen™, A7592) for 2 hours. Cells were washed with media and treated with 2 mM of Furd (Sigma, F5130-100 mg) for 10 min. All incubations were performed in the humidified CO_2_ incubator. To stop the assay, cells were washed with ice-cold PBS, fixed with 3.7% formaldehyde, permeabilized with 0.5% Triton, and blocked in 0.5% milk overnight at 4°C. Then, cells were stained with primary anti-BrdU antibodies (B2531, Sigma) 1:50 for 1 h and secondary Alexa488 antibodies (Jackson Immuno Research, 715-545-151) 1:400 for 1 h. Coverslips were mounted using VECTASHIELD® Hardset™ Antifade Mounting Medium with DAPI (Vector Laboratories, H-1500-10) and imaged on the confocal microscope Leica STED.

### Immunohistochemical staining (IHC)

Skin Melanoma Tissue Microarray (TMA) (US Biolab, MEL0100-02A) was deparaffinized and rehydrated in xylene followed by 100%, 95%, 70%, and 50% ethanol. Antigen retrieval was performed for 20 min, 100 ⁰C in the sodium-citrate buffer. IHC staining was performed using the mouse and rabbit-specific HRP/DAB detection IHC kit (Abcam, ab64264) according to the manufacturer’s instructions. Briefly, blocking with a protein block solution was followed by primary antibody incubation, then biotinylated goat anti-polyvalent was applied. After 4 washes, streptavidin peroxidase was applied, and after incubation slide was stained with DAB and counterstained with hematoxylin.

Tumor samples collected from C3H mice were fixed in buffered formalin for 48 h with subsequent ethanol fixation for 48 h. Paraffin-embedded samples were processed on the Leica Bond staining system using BOND Polymer refine detection kit (DS9800) according to the manufacturer’s protocol for the corresponding antibody.

Information about antibodies is provided in the Supplementary Materials and Methods Table S5. Images were captured using Leica Versa 8 microscope.

Quantification was performed using Fiji software. Color deconvolution into DAB and Hematoxylin channels was performed. % of POLR1A positive area was calculated as a ratio of the area stained with DAB to the area stained with hematoxylin, indicating the total area of tissue. For the evaluation of the number of CD8-positive cells, a manual count was performed in 5 randomly selected fields of view (3 on the tumor edges and 2 in the central part).

### Polysome profiling

Polysome profiling was performed according to the previously published protocols (9,10) . Briefly, cells at 80% confluency were treated with 100 ug/mL of cycloheximide, lysed, and then 2 mg of protein was loaded on top of the sucrose gradient. Gradients were spun in a SW41Ti rotor at 32,000rpm for 3.5 hours at 4 ⁰C. 10 fractions were collected using the fractionator, RNA was precipitated overnight in -80 ⁰C with three volumes 100% ethanol added to each fraction. The next day precipitate was centrifuged at 16,000g for 20 min and the supernatant was removed, with subsequent RNA isolation from the pellet using the Trizol according to the manufacturer’s instructions. RT was performed using the iScript™ cDNA Synthesis Kit (Biorad, 1708890) and qPCR was set up using iTaq™ Universal SYBR® Green Supermix (Biorad, 1725121). Calculations for % of mRNA in each monosome or polysome fraction were performed according to the previously published method (10). For the graph creation, a spline curve was generated using Graphpad.

**References**

1. J E Talmadge IJF. Enhanced metastatic potential of tumor cells harvested from spontaneous metastases of heterogeneous murine tumors - PubMed. J Natl Cancer Inst . 1982 Oct;69(4)(975–80).

2. Elcheva I, Brok-Volchanskaya V, Slukvin I. Direct Induction of Hemogenic Endothelium and Blood by Overexpression of Transcription Factors in Human Pluripotent Stem Cells. J Vis Exp. 2015 Dec 3;2015(106):52910.

3. Akbani R, Akdemir KC, Aksoy BA, Albert M, Ally A, Amin SB, et al. Genomic Classification of Cutaneous Melanoma. Cell. 2015 Jun 20;161(7):1681.

4. Schneider CA, Rasband WS, Eliceiri KW. NIH Image to ImageJ: 25 years of image analysis. Nat Methods. 2012 Jul 28;9(7):671–5.

5. Borowicz S, Van Scoyk M, Avasarala S, Karuppusamy Rathinam MK, Tauler J, Bikkavilli RK, et al. The Soft Agar Colony Formation Assay. J Vis Exp. 2014;(92):51998.

6. Zhong Y, Karaletsos T, Drewe P, Sreedharan VT, Kuo D, Singh K, et al. RiboDiff: detecting changes of mRNA translation efficiency from ribosome footprints. Bioinformatics. 2016 Jan 1;33(1):139.

7. Schindelin J, Arganda-Carreras I, Frise E, Kaynig V, Longair M, Pietzsch T, et al. Fiji - an Open Source platform for biological image analysis. Nat Methods. 2012 Jul;9(7):10.1038/nmeth.2019.

8. Percipalle P, Louvet E. In vivo run-on assays to monitor nascent precursor RNA transcripts. Methods Mol Biol. 2012;809:519–33.

9. Morita M, Alain T, Topisirovic I, Sonenberg N. Polysome Profiling Analysis. Bio Protoc. 2013 Jul 20;3(14).

10. Panda AC, Martindale JL, Gorospe M. Polysome Fractionation to Analyze mRNA Distribution Profiles. Bio Protoc [Internet]. 2017 [cited 2025 Dec 14];7(3):e2126. Available from: https://pmc.ncbi.nlm.nih.gov/articles/PMC5431591/
